# The Relationship between 25 (OH) D Levels (Vitamin D) and Bone Mineral Density (BMD) in a Saudi Population in a Community-Based Setting

**DOI:** 10.1371/journal.pone.0169122

**Published:** 2017-01-03

**Authors:** Abdullah Alkhenizan, Ahmed Mahmoud, Aneela Hussain, Alia Gabr, Suad Alsoghayer, Abdelmoneim Eldali

**Affiliations:** 1 Department of Family Medicine, King Faisal Specialist Hospital and Research Centre, Riyadh, Kingdom of Saudi Arabia; 2 Department of Biostatistics, Epidemiology and Scientific Computing, King Faisal Specialist Hospital and Research Centre, Riyadh, Kingdom of Saudi Arabia; University of Alabama at Birmingham, UNITED STATES

## Abstract

**Background:**

Vitamin D deficiency has been linked to an increased risk of osteoporosis. Vitamin D deficiency has reached high levels in the Saudi population, but there is conflicting evidence both in the Saudi population, and worldwide, regarding the existence of a correlation between these low vitamin D levels and reduced BMD (bone mineral density), or osteoporosis.

**Objective:**

The objective of this study was primarily to determine whether there was a correlation between vitamin D deficiency and osteoporosis in the Saudi population. We aimed to investigate whether the high levels of vitamin D deficiency and insufficiency would translate to higher prevalence of osteoporosis, and whether there is a correlation between vitamin D levels and bone mineral density.

**Materials and methods:**

This was a community based cross sectional study conducted in the Family Medicine Clinics at King Faisal Specialist Hospital and Research Centre in Riyadh, Saudi Arabia. Electronic records of 1723 patients were reviewed. Laboratory and radiology results were collected, including vitamin D levels, calcium levels, and bone mineral density scan results.

**Results:**

Among the whole population, 61.5% had moderate to severe vitamin D deficiency with levels less than 50nmol/L. 9.1% of the population had osteoporosis, and 38.6% had osteopenia. Among the whole population, there was no significant correlation between spine or total femoral BMD and serum 25(OH) D.

**Conclusion:**

Vitamin D deficiency is prevalent in the Saudi population. However, no correlation has been found between vitamin D deficiency and reduced bone mineral density in any age group, in males or females, Saudis or Non-Saudis, in our population in Riyadh, Saudi Arabia.

## Introduction

Vitamin D deficiency has been linked to secondary hyperparathyroidism and bone loss, with reduction in BMD and an increased risk of osteoporosis [1,2]. The general consensus on vitamin D deficiency suggests that deficiency exists when serum 25-hydroxyvitamin D levels are less than 50nmol/L, with insufficiency in the range from 50nmol/L to 75nmol/L, and sufficiency above 75nmol/L [3].

Vitamin D status is assessed by measurement of 25-Hydroxyvitamin D in the blood. Maintaining recommended levels of vitamin D is essential for bone health. Sources of vitamin D include adequate exposure to sunlight and sufficient intake of vitamin D from dietary sources. Supplements should be used if the vitamin D level is insufficient [3].

According to one of the largest and most recent studies on vitamin D status in the population, 83.6% of the study population was vitamin D deficient. The Middle East has the highest rates of vitamin D deficiency worldwide, despite its geography [4]. These studies show the importance of understanding the effects of vitamin D deficiency on our local population. Clearly the prevalence of the deficiency is startling in the Saudi Arabian population and this should lead to an increased prevalence of osteoporosis, then we might expect a significant increase in fractures and worsening bone health. As such, we conducted this study to investigate whether such a high prevalence of vitamin D deficiency is correlated with an increased prevalence of osteoporosis.

In this study we assessed the relationship between vitamin D levels and BMD within the Saudi population in order to give us a more complete picture of factors affecting bone health in our locality, and assist us in providing the most suitable care. Three previous studies looked at the link between vitamin D and BMD in our local population in the Kingdom of Saudi Arabia [5,6,7]. However, those previous studies were smaller in size. Our significantly larger sample size gives us more power to investigate and test any association between BMD and vitamin D levels. In addition, our study is more community based, as it was carried out at the primary care level.

There is certainly a need for further, large, well-constructed studies to try to clear up the controversy surrounding this issue. With this study, we hoped to increase the understanding of the link between osteoporosis and vitamin D levels in our local population, and take another step towards a clearer picture of this controversial topic.

## Materials and Methods

Ethical considerations: The research project was conducted in accordance with the guiding principles for experimental procedures written in the Declaration of Helsinki of the World Medical Association, and the NIH Guide for the Care and Use of Laboratory Animals, 1985, contained in the Declaration of Helsinki (2000) and is approved by the institutional review board of KFSH&RC. It complies with the policies of the Research Advisory Committee (RAC) at KFSH&RC and the laws of the Kingdom of Saudi Arabia. RAC# 2151–125.

This study was conducted in the Family Medicine Department at King Faisal Specialist Hospital and Research Centre (KFSH&RC) in Riyadh, Saudi Arabia. The department of Family Medicine provides primary care services to a catchment population of 50,000 within the community. The electronic health records of 1723 patients who had BMD screening and vitamin D levels documented were reviewed retrospectively. The demographic data and laboratory results were collected, including 25-Hydroxy vitamin D levels, BMD, albumin, phosphate, and calcium levels. All the statistical analysis of data was done by using the software package SPSS, version 20.0 by IBM. Descriptive statistics for the continuous variables are reported as median and mean, and categorical variables are summarized as frequencies and percentages. Categorical variables are compared by use of the Chi-squared test. The level of statistical significance is set at p < 0.05. Pearson correlation was used to test the correlation between BMD and 25 (OH) D.

## Results

A total number of 1723 patients were enrolled in this study. 84.3% (n = 1452) of the study population were females and 15.7% (n = 271) were males. When we stratify the study population by nationality, we have 69.2% (n = 1193) Saudi and 30.8% (n = 530) Non-Saudi. Looking at age groups among the study population, we found 34.4% (n = 593) elderly (60 years and older) and 65.6% (n = 1130) adult. Among the whole population, 24.8% (n = 427) have severe vitamin D deficiency with 25-hydroxyvitamin D levels less than 25nmol/L, 36.7% (n = 633) have moderate vitamin D deficiency with serum 25-hydroxyvitamin D levels between 25nmol/L and 50nmol/L, 20.7% (n = 356) have mild vitamin D deficiency with serum 25-hydroxyvitamin D levels between 50nmol/L and 75nmol/L, while only 17% (n = 302) have optimum levels of serum 25-hydroxyvitamin D above 75nmol/L (Table 1 & S1 Fig). The T-total score showed that 9.1% (n = 157) of the population had osteoporosis, 38.6% (n = 665) had osteopenia, and 52.3% (n = 901) were normal. Among the whole population, there was no significant correlation between spine or total femoral BMD and serum 25(OH) (Table 2).

**Table 1:** Overview of study population.

Total No. of Participants = 1723		
**Parameters**	**No.**	**Percentage**
**Nationality**		
Saudi	1193	69.2%
Non-Saudi	530	30.8%
**Gender**		
Male	271	15.7%
Female	1452	84.3%
**Age Group**		
Adult	1130	65.6%
Elderly (60+)	593	34.4%
**T-total**		
Normal	901	52.3%
Osteopenia	665	38.6%
Osteoporosis	157	9.1%
**VD deficiency**		
Optimum	302	17.5%
Mild	356	20.7%
Moderate	633	36.7%
Severe	427	24.8%

**Table 2:** Correlation between vitamin D and BMD.

		T-spine	P- value	T-neck	P-value
Vitamin D (D 25-Hydroxy)	All population	-.030	.221	-.070*	.005
	Male	-.042	.492	-.085	.173
	Female	-.026	.316	-.054*	.046

* Correlation is significant at the 0.05 level (2-tailed).

In addition, we stratified the participants into three groups, according to their 25(OH)D levels, those with levels of 75nmol/l and above, those with levels 50nmol/l and above, and finally those with levels 25nmol/l and below. We did not find any significant correlation between spine or total femoral BMD and serum 25(OH)D in any of these groups (S1 Tables).

Furthermore, using the Chi-squared test, we did not find any statistically significant association between BMD and vitamin D levels (>75nmol/l vs. <25nmol/l), nor with BMD and vitamin D levels (>50nmol/l vs. <25nmol/l).

### Female subset

1452 females were included in this study. 68.5% (n = 995) of them were Saudis and 31.5% (n = 457) were Non-Saudis. The T-total score was normal in 55% (n = 798) of the females, 36.4% (n = 529) had osteopenia and 8.6% (n = 125) had osteoporosis (S2 Fig). Using chi-square test, age and nationality have significant association with T-score with (p<0.0001). The prevalence of osteoporosis among Saudis was 10.3% (n = 102) and 5.0% (n = 23) among non-Saudis (p<0.0001). When we compared women by age group, we found that the prevalence of osteoporosis among elderly females was 18.8% (87 of 464), while only 3.8% (38 of 988) of adult females had osteoporosis (p<0.0001). Both spine and total femoral BMD failed to show any significant correlation with serum 25(OH) D in any of the age groups in Saudi or Non-Saudi females (Table 3). The female subset was also divided into premenopausal (age 21–49), and postmenopausal (age >50) women. No significant correlation between spine or total femoral BMD and serum 25(OH)D was found in either of these groups. Similarly, this subset was divided into elderly (60 years or above), and non-elderly (<60 years), and again no statistically significant correlation was found in either group (S1 Tables).

**Table 3:** Gender analysis.

Total No. of Participants = 1723		
**Parameters**	**Male (N = 271)**	**Female (N = 1452)**
**Nationality**		
Saudi	73.1%	68.5%
Non-Saudi	26.9%	31.5%
**Age Group**		
Adult	52.3%	68%
Elderly (60+)	47.6%	32%
**T-total**		
Normal	38%	55%
Osteopenia	50.2%	36.4%
Osteoporosis	11.8%	8.6%
**VD deficiency**		
Optimum	18.1%	17.4%
Mild	22.5%	20.3%
Moderate	38.7%	33.9%
Severe	20.3%	28.1%

### Male subset

We had 271 males included in this study. 73.1% (n = 198) of them were Saudis and 26.9% (n = 73) were Non-Saudis. The T-total score was normal in 38% (n = 103) of the males, whereas 50.2% (n = 136) had osteopenia, and 11.8% (n = 32) had osteoporosis (S2 Fig). Using chi-square test, age has significant association with osteopenia and osteoporosis. Osteoporosis prevalence was 18.6% (24 of 129) among elderly males, while only 5.6% (8 of 142) of adult males were diagnosed with osteoporosis, (p<0.0001). We did not find any significant correlation between both spine and total femoral BMD with serum 25(OH) D in any age group, in Saudi or Non-Saudi males (Table 3).

## Discussion

The most pertinent findings of this study are that analysis of the data showed no significant correlation between vitamin D and Bone Mineral Density in any age groups, in males or females. These findings are in agreement with one previous Saudi study [7], but not the two studies by Ardawi et al, which showed significant correlation between vitamin D levels and BMD [5,6]. The causes of the discrepancies are not clear, but some confounding factors which may have contributed include the fact that the two studies which found positive correlation were located on the west coast of Saudi Arabia, in the city of Jeddah, whereas our study was focused on patients in Riyadh in the central desert region, so there are geographical differences which contribute to differences in environment, climate and local customs. As an example Riyadh’s climate is generally hotter in summer, but cooler in winter, while Jeddah is significantly more humid. In addition, Jeddah is a coastal town and the diet will reflect that, with higher consumption of seafood. The other Riyadh study also found no significant correlation between vitamin D levels and BMD [7]. There are also differences in patient populations, with the Jeddah studies concentrating on Saudi Arabian nationals only. Our patient population was drawn from community based family health clinics in Riyadh. These included both Saudi and Non-Saudi patients. According to the census of Saudi Arabia, 33% of the population is non-Saudi nationals, which corresponds closely to the non-Saudi population in our study with 30.8% of non-Saudi participants [8]. However, when we split up the patient population into Saudi and Non-Saudi groups, we still were unable to find any correlation between vitamin D levels and BMD at any site.

Among the female subset, we found that there was a significant association between T-score and both age and nationality. Saudi women were more than twice as likely to have osteoporosis as non-Saudi women. This could be due to several factors including lifestyle differences, physical activity levels, direct sunlight exposure, and the fact that Saudi women are more likely to wear Arab/Muslim style dress with full skin covering. The same nationality differences were not found in the male subset, where Saudi men were no more likely to suffer from osteoporosis than non-Saudi men. Similarly, as expected, elderly females were much more likely than adult females to have osteoporosis, with the same applying to elderly males who were also much more likely to have osteoporosis.

Vitamin D has a role to play in the maintenance of good bone health, and this has been well documented in numerous investigations in a variety of locations, but the presence of a direct correlation between vitamin D levels (1, 25 dihydroxyvitamin D) and bone mineral density is still a controversial issue, with some investigators observing a significant correlation, and others unable to find any correlation [5,7,9,10,11].

Even when we looked for an association by comparing BMD prevalence in three separate groups, according to vitamin D levels, (<25nmol/l vs. >25nmol/l vs. >75nmol/l), we were still unable to find any statistically significant difference in the prevalence of osteoporosis or osteopenia in these groups, and no significant correlation between vitamin D and BMD in any of these groups. So no significant association was found.

Reviewing previous studies on this subject, both Saudi and international, it would appear that there are a multitude of variables which have an impact on the presence or absence of a correlation. These include, but are not limited to, age, sex, geographical location, hormonal status and ethnicity/race. For post-menopausal women, a Japanese study in 2007 failed to find a significant association between serum 25(OH) D and BMD of the lumber spine, but did find a positive association between serum 25(OH) D and BMD of the femoral neck [9]. The Saudi study on postmenopausal women with osteoporosis found a significant positive correlation between serum 25(OH) D and BMD of the femoral neck in postmenopausal women with osteoporosis [5]. When we split our female subset into pre and postmenopausal groups, again we were unable to find any significant correlation in either group.

Focusing on the male population, a separate Saudi study found a positive correlation between serum 25 (OH) D and BMD of both the lumbar spine (p<0.023) and femoral neck (p<0.036) in healthy Saudi Arabian men [6]. Racial variations were highlighted by a diverse study in 2008 by Hannan et al, focusing on men from a variety of racial and ethnic backgrounds. Interestingly, a significant positive correlation between 25 (OH) D levels and BMD was found in White men only, while it was not significant in Hispanic or Afro-Caribbean men [12].

Conversely, many studies in both genders did not find any significant correlation between BMD and 25 (OH) D. A recent large Chinese study (2015) with a sample size of over 10000, did not find a significant correlation between 25 (OH) D and BMD in North-Western China [10]. The study also showed that poor serum 25 (OH) D concentrations did not increase the risk of osteoporosis. The same result was found in a study done in Morocco, which showed no significant correlation between spinal or femoral BMD and 25 (OH) D levels in both genders [13]. A further study looking into the relationship between BMD and 25-Hydroxyvitamin D3 in Postmenopausal Iranian Women in 2001, again failed to find a significant correlation between hip BMD and serum 25-OHD3. However, serum 25-OHD3 was found to have a weak correlation with lumbar spine BMD [11]. Similarly, a study among healthy Saudi women did not find any correlation between BMD values and 25 (OH) D levels [7].

Finally, a double blind randomized controlled global trial of postmenopausal women by Lips et al in 2001, with a large sample size, found that the effect of vitamin D deficiency on BMD was small and not significant, except for BMD of the trochanter, where a serum vitamin D level below 25nmol/l was associated with a 4% lower BMD [14].

In conclusion, we were unable to find any significant correlation between BMD and vitamin D in our patient population. There appear to be many factors that have an impact on whether correlation exists as discussed. In addition, calcium intake may be a modifying factor as well as vitamin D supplementation. Clearly with a cross-sectional study like this one, it is quite difficult to assess the extent of calcium intake and vitamin D supplementation among our patient population, and therefore its possible effect on the outcome of our study. Another issue with our cross-sectional study is the fact that in our primary care clinics, the BMD scan for males would only have been requested on higher risk patients who are more likely to have osteopenia or osteoporosis, or those who are candidates for screening. As such, the male population we captured might not give us a true normal range, and may skew results towards a higher prevalence of osteoporosis.

Another confounding factor could be the fact that there may well be other vitamin D derivatives or metabolites, which are biologically active, but not reflected in the vitamin D levels measured in our laboratories. Analysis of human serum and epidermis has revealed the presence of 20(OH)D3, 22(OH)D3 and a whole host of other vitamin D derivatives which are likely to be biologically active [15]. This information might call into question the accuracy of our current methods used to estimate total active vitamin D levels in the body.

Despite the sundrenched climate in the Kingdom of Saudi Arabia, we find that the prevalence of vitamin D deficiency and insufficiency is strikingly high. Osteoporosis is an important public health concern, and the more advanced our understanding of the role of vitamin D in the disease process, the more effective our strategies will be for the local prevention of this disease, particularly since vitamin D deficiency is preventable.

## Supporting Information

S1 Dataset Complete study dataset. (XLSX)

S1 Fig Vitamin D deficiency across genders. (PDF)

S2 Fig BMD across genders. (PDF)

S1 Tables Supplemental Data analysis. (DOCX)
