# Supplementary figures and images for: The Relationship between 25 (OH) D Levels (Vitamin D) and Bone Mineral Density (BMD) in a Saudi Population in a Community-Based Setting
Source: PLoS One. 2017 Jan 3;12(1):e0169122. doi: 10.1371/journal.pone.0169122 (PMC5207714; doi:10.1371/journal.pone.0169122)

## VD deficiency among Genders

Male Female

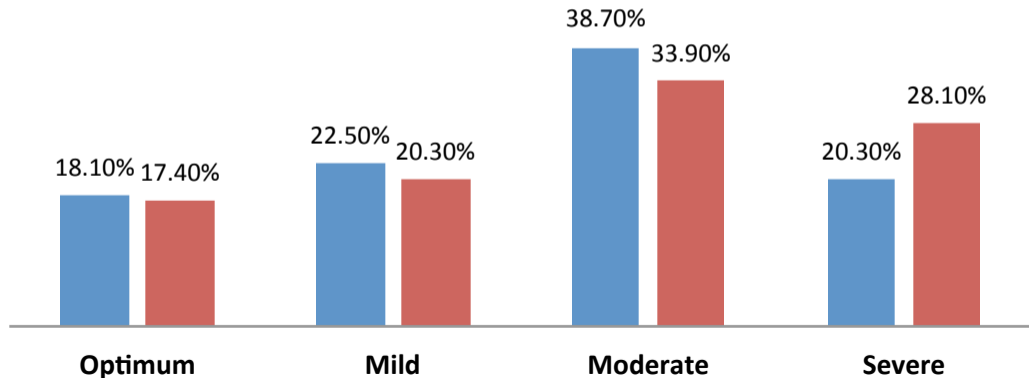

Supplement: S1 Fig — (PDF) [file pone.0169122.s002.pdf]

# T-Total Score among Genders

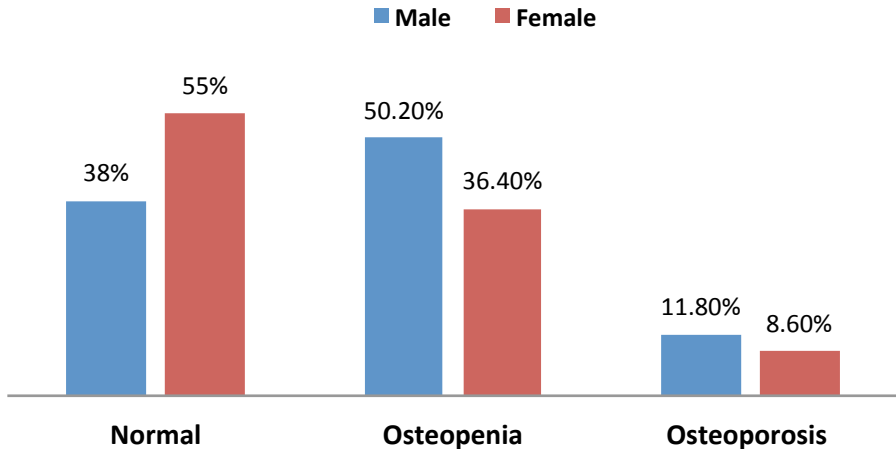

Supplement: S2 Fig — (PDF) [file pone.0169122.s003.pdf]
