# Supplementary material for: The Relationship between 25 (OH) D Levels (Vitamin D) and Bone Mineral Density (BMD) in a Saudi Population in a Community-Based Setting
Source: PLoS One. 2017 Jan 3;12(1):e0169122. doi: 10.1371/journal.pone.0169122 (PMC5207714; doi:10.1371/journal.pone.0169122)
Supplement: S1 Tables — (DOCX) [file pone.0169122.s004.docx]

**Supplemental Data Analysis**

*Data Analysis:*

- The prevalence of osteoporosis/osteopenia in the whole population of women and men with 25(OH)D 75 and greater nmol/L

| **T-total** | | | | | |
| --- | --- | --- | --- | --- | --- |
|  | | Frequency | Percent | Valid Percent | Cumulative Percent |
| Valid | Normal | 154 | 50.2 | **50.2** | 50.2 |
|  | Osteopenia | 117 | 38.1 | **38.1** | 88.3 |
|  | Osteoporosis | 36 | 11.7 | **11.7** | 100.0 |
|  | Total | **307** | 100.0 | 100.0 |  |

- The prevalence of osteoporosis/osteopenia in **men** with 25(OH)D 75 and greater nmol/L

| **T-total** | | | | | |
| --- | --- | --- | --- | --- | --- |
|  | | Frequency | Percent | Valid Percent | Cumulative Percent |
| Valid | Normal | 21 | 42.0 | **42.0** | 42.0 |
|  | Osteopenia | 17 | 34.0 | **34.0** | 76.0 |
|  | Osteoporosis | 12 | 24.0 | **24.0** | 100.0 |
|  | Total | **50** | 100.0 | 100.0 |  |

- The prevalence of osteoporosis/osteopenia in **women** with 25(OH)D 75 and greater nmol/L

| **T-total** | | | | | |
| --- | --- | --- | --- | --- | --- |
|  | | Frequency | Percent | Valid Percent | Cumulative Percent |
| Valid | Normal | 133 | 51.8 | **51.8** | 51.8 |
|  | Osteopenia | 100 | 38.9 | **38.9** | 90.7 |
|  | Osteoporosis | 24 | 9.3 | **9.3** | 100.0 |
|  | Total | **257** | 100.0 | 100.0 |  |

**Summary of correlations between Vitamin D and BMD (vit D>75)**

|  | | T-spine | P- value | T-neck | P-value |
| --- | --- | --- | --- | --- | --- |
| Vitamin D  (D 25-Hydroxy) | All population VD≥75 | -.055 | .334 | -.026 | .675 |
|  | Male VD≥75 | -.125 | .389 | -.138 | .365 |
|  | Female VD≥75 | -.017 | .789 | .063 | .348 |

- The prevalence of osteoporosis/osteopenia in the whole population of women and men with 25(OH)D 50 and greater nmol/L

| **T-total** | | | | | |
| --- | --- | --- | --- | --- | --- |
|  | | Frequency | Percent | Valid Percent | Cumulative Percent |
| Valid | Normal | 332 | 50.1 | **50.1** | 50.1 |
|  | Osteopenia | 265 | 40.0 | **40.0** | 90.0 |
|  | Osteoporosis | 66 | 10.0 | **10.0** | 100.0 |
|  | Total | **663** | 100.0 | 100.0 |  |

- The prevalence of osteoporosis/osteopenia in **men** with 25(OH)D 50 and greater nmol/L

| **T-total** | | | | | |
| --- | --- | --- | --- | --- | --- |
|  | | Frequency | Percent | Valid Percent | Cumulative Percent |
| Valid | Normal | 42 | 37.8 | **37.8** | 37.8 |
|  | Osteopenia | 53 | 47.7 | **47.7** | 85.6 |
|  | Osteoporosis | 16 | 14.4 | **14.4** | 100.0 |
|  | Total | **111** | 100.0 | 100.0 |  |

- The prevalence of osteoporosis/osteopenia in **women** with 25(OH)D 50 and greater nmol/L

| **T-total** | | | | | |
| --- | --- | --- | --- | --- | --- |
|  | | Frequency | Percent | Valid Percent | Cumulative Percent |
| Valid | Normal | 290 | 52.5 | **52.5** | 52.5 |
|  | Osteopenia | 212 | 38.4 | **38.4** | 90.9 |
|  | Osteoporosis | 50 | 9.1 | **9.1** | 100.0 |
|  | Total | **552** | 100.0 | 100.0 |  |

**Summary of correlations between Vitamin D and BMD (vit D>50)**

|  | | T-spine | P- value | T-neck | P-value |
| --- | --- | --- | --- | --- | --- |
| Vitamin D  (D 25-Hydroxy) | All population VD≥50 | -.033 | .394 | -.019 | .645 |
|  | Male  VD≥50 | -.087 | .362 | -.104 | .288 |
|  | Female  VD≥50 | -.009 | .828 | .028 | .530 |

- The prevalence of osteoporosis/osteopenia in the whole population of women and men with 25(OH)D 25nmol/L and below

| **T-total** | | | | | |
| --- | --- | --- | --- | --- | --- |
|  | | Frequency | Percent | Valid Percent | Cumulative Percent |
| Valid | Normal | 261 | 56.4 | **56.4** | 56.4 |
|  | Osteopenia | 167 | 36.1 | **36.1** | 92.4 |
|  | Osteoporosis | 35 | 7.6 | **7.6** | 100.0 |
|  | Total | **463** | 100.0 | 100.0 |  |

- The prevalence of osteoporosis/osteopenia in **men** with 25(OH)D 25nmol/L and below

| **T-total** | | | | | |
| --- | --- | --- | --- | --- | --- |
|  | | Frequency | Percent | Valid Percent | Cumulative Percent |
| Valid | Normal | 22 | 40.0 | **40.0** | 40.0 |
|  | Osteopenia | 27 | 49.1 | **49.1** | 89.1 |
|  | Osteoporosis | 6 | 10.9 | **10.9** | 100.0 |
|  | Total | **55** | 100.0 | 100.0 |  |

- The prevalence of osteoporosis/osteopenia in **women** with 25(OH)D 25nmol/L and below

| **T-total** | | | | | |
| --- | --- | --- | --- | --- | --- |
|  | | Frequency | Percent | Valid Percent | Cumulative Percent |
| Valid | Normal | 239 | 58.6 | **58.6** | 58.6 |
|  | Osteopenia | 140 | 34.3 | **34.3** | 92.9 |
|  | Osteoporosis | 29 | 7.1 | **7.1** | 100.0 |
|  | Total | **408** | 100.0 | 100.0 |  |

**Summary of correlations between Vitamin D and BMD (vit D<25)**

|  | | T-spine | P- value | T-neck | P-value |
| --- | --- | --- | --- | --- | --- |
| Vitamin D  (D 25-Hydroxy) | All population VD≤ 25 | -.045 | .337 | -.095 | .049* |
|  | Male VD≤ 25 | .163 | .236 | .111 | .442 |
|  | Female VD≤ 25 | -.054 | .278 | -.093 | .068 |

- The prevalence of osteoporosis/osteopenia in the **elderly** population

| **T-total** | | | | | |
| --- | --- | --- | --- | --- | --- |
|  | | Frequency | Percent | Valid Percent | Cumulative Percent |
| Valid | Normal | 184 | 31.0 | **31.0** | 31.0 |
|  | Osteopenia | 298 | 50.3 | **50.3** | 81.3 |
|  | Osteoporosis | 111 | 18.7 | **18.7** | 100.0 |
|  | Total | **593** | 100.0 | 100.0 |  |

- The prevalence of osteoporosis/osteopenia in elderly **men**

| **T-total** | | | | | |
| --- | --- | --- | --- | --- | --- |
|  | | Frequency | Percent | Valid Percent | Cumulative Percent |
| Valid | Normal | 38 | 29.5 | **29.5** | 29.5 |
|  | Osteopenia | 67 | 51.9 | **51.9** | 81.4 |
|  | Osteoporosis | 24 | 18.6 | **18.6** | 100.0 |
|  | Total | **129** | 100.0 | 100.0 |  |

- The prevalence of osteoporosis/osteopenia in elderly **women**

| **T-total** | | | | | |
| --- | --- | --- | --- | --- | --- |
|  | | Frequency | Percent | Valid Percent | Cumulative Percent |
| Valid | Normal | 146 | 31.5 | **31.5** | 31.5 |
|  | Osteopenia | 231 | 49.8 | **49.8** | 81.3 |
|  | Osteoporosis | 87 | 18.8 | **18.8** | 100.0 |
|  | Total | **464** | 100.0 | 100.0 |  |

**Summary of correlations between Vitamin D and BMD (Elderly)**

|  | | T-spine | P- value | T-neck | P-value |
| --- | --- | --- | --- | --- | --- |
| Vitamin D  (D 25-Hydroxy) | All elderly population | .017 | .678 | -.013 | .761 |
|  | Elderly men | -.090 | .311 | -.095 | .308 |
|  | Elderly women | .072 | .119 | .051 | .291 |

• The prevalence of osteoporosis/osteopenia in all **women**

| **T-total** | | | | | |
| --- | --- | --- | --- | --- | --- |
|  | | Frequency | Percent | Valid Percent | Cumulative Percent |
| Valid | Normal | 798 | 55.0 | **55.0** | 55.0 |
|  | Osteopenia | 529 | 36.4 | **36.4** | 91.4 |
|  | Osteoporosis | 125 | 8.6 | **8.6** | 100.0 |
|  | Total | **1452** | 100.0 | 100.0 |  |

• The prevalence of osteoporosis/osteopenia in **premenopausal** women (age 21-49)

| **T-total** | | | | | |
| --- | --- | --- | --- | --- | --- |
|  | | Frequency | Percent | Valid Percent | Cumulative Percent |
| Valid | Normal | 285 | 74.2 | **74.2** | 74.2 |
|  | Osteopenia | 94 | 24.5 | **24.5** | 98.7 |
|  | Osteoporosis | 5 | 1.3 | **1.3** | 100.0 |
|  | Total | **384** | 100.0 | 100.0 |  |

• The prevalence of osteoporosis/osteopenia in **menopausal** women (age 50+)

| **T-total** | | | | | |
| --- | --- | --- | --- | --- | --- |
|  | | Frequency | Percent | Valid Percent | Cumulative Percent |
| Valid | Normal | 513 | 48.0 | **48.0** | 48.0 |
|  | Osteopenia | 435 | 40.7 | **40.7** | 88.8 |
|  | Osteoporosis | 120 | 11.2 | **11.2** | 100.0 |
|  | Total | **1068** | 100.0 | 100.0 |  |

**Summary of correlations between Vitamin D and BMD (Women)**

|  | | T-spine | P- value | T-neck | P-value |
| --- | --- | --- | --- | --- | --- |
| Vitamin D  (D 25-Hydroxy) | All women | -.026 | .316 | -.054* | .046 |
|  | Premenopausal women | -.052 | .314 | -.050 | .344 |
|  | Menopausal women | .011 | .711 | -.028 | .378 |
